# Anatomical Insights Into Profound Pulmonary Venous Malformation in an Elderly Individual: A Report of a Rare Cadaveric Case

**DOI:** 10.7759/cureus.87905

**Published:** 2025-07-14

**Authors:** Madeleine Schwab, Samantha Spence, Sophia Izhar, Andrey Frolov, Yun Tan, Daniel T Daly

**Affiliations:** 1 Department of Surgery - Center for Anatomical Science and Education, Saint Louis University School of Medicine, Saint Louis, USA

**Keywords:** accessory pulmonary veins, embryology, pulmonary abnormalities, pulmonary vein variations, vessel anomalies

## Abstract

A male cadaver, 87 years of age, was received through the Saint Louis University Gift of Body Program. Significant pulmonary vein (PV) variations were observed following routine dissection. The donor displayed two normal left PVs and 10 significantly undersized right PVs. By caliper measurement, the left PVs had long axis diameters of 19 mm and 20 mm, while the right PVs ranged from 1 mm to 11 mm. The total cross-sectional area of the 10 right PVs was 173 mm^2^ which was significantly less than the 429 mm^2^ total cross-sectional area of the two left PVs. This was unexpected since the total diameter and cross-sectional area of right PVs are usually reported to be larger. While it is not uncommon for patients to have slight variations in the pulmonary venous structure, there are no known reports of individuals with 12 asymmetric PVs. Anatomical variation in the quantity and dimensions of PVs results in abnormal blood drainage and increased fluid resistance in the vessels, which could potentially contribute to cardiopulmonary sequelae. Appreciation of variations and their possible developmental underpinnings could allow for a better understanding of pathology and appropriate treatment for patients with anomalous vessels and cardiovascular diseases. For example, recent correlations between PV defects, atrial fibrillation, and ectopic heartbeats have been elucidated. Individuals with abnormal PVs have been found to express higher rates of ectopic foci resulting in atrial fibrillation. Successful resolution of ectopic foci via radiofrequency catheter ablation requires accessory PVs to be effectively identified and accounted for when evaluating treatment options. As such, a comprehensive understanding of both typical and atypical pulmonary vasculature prompts refinement of interventional procedures for maximal safety and efficacy for these patients.

## Introduction

Human lungs are the main organ responsible for gas exchange. Deoxygenated blood is delivered to the lungs via the pulmonary arteries, and oxygenated blood is returned to the left atrium following gas exchange via the pulmonary veins (PVs) before being distributed systemically through the aorta and its branches. 

Typically, each lung is associated with two PVs, both positioned anterior and inferior to the pulmonary arteries, that pass oxygenated blood back to the heart. This creates four ostia in the left atrium: one each for the left superior PV, left inferior PV, right superior PV, and right inferior PV. The superior PVs collect blood from the right upper and middle lobes and the left upper lobe. On both sides, the inferior PVs drain the lower lobes. Notably, the venous structure of the pulmonary circulation is not a simple mirror image of the pulmonary artery branching within the lungs [1]. There are two tributaries of each PV in each bronchopulmonary segment: the intrasegmental vein and the infrasegmental vein. The intrasegmental branch runs with the bronchus and the pulmonary artery, whereas the infrasegmental branch travels along the inferior border of the segment. The infrasegmental veins serve as the boundaries of the bronchopulmonary segments and are used as landmarks for resection of the bronchopulmonary segments [2]. PVs are an essential component of the circulatory system, and a comprehensive understanding of their normal development and function is integral for medical knowledge. 

In normal embryological development, the lungs form from the lung bud, a diverticulum that forms along the ventral aspect of the foregut at the laryngotracheal groove. The lung buds share a common vascular plexus, the splanchnic plexus, which initially drains into the common cardinal and umbilicovitelline venous systems. A portion of the splanchnic plexus will eventually differentiate to become the primitive pulmonary vascular bed. The common pulmonary vein (CPV), or primitive PV, derived from mediastinal myocardium, simultaneously forms from a caudal outpouching of the primitive left atrium and extends towards the newly formed lung bud [3]. At 28 days gestation, the CPV joins the pulmonary portion of the splanchnic plexus. The CPV opens initially as a solitary orifice adjacent to the atrioventricular junctions with four tributaries [4]. As the left atrium grows, the CPV merges with the atrial wall, forming its smooth portion. The four primitive veins are then formed from the CPV tributaries, and the connections with the cardinal and umbilicovitelline veins are obliterated, leaving four independent PVs draining directly into the left atrium through their own individual ostia [5]. Abnormal resorption of the embryonic structures can lead to alterations in the diameter and number of PVs [6]. 

Notably, the expected pattern of two ostia each on the right and left sides of the left atrium only occurs in 70% of the population [7]. The most common variances include the presence of a single PV on one side or an additional PV, resulting in the presence of three veins in a single lung hilum [8]. In addition to supernumerary PVs, there are unusual venous drainage anomalies, such as partial anomalous pulmonary venous return (PAPVR), where one or more PVs carry blood to the right side of the heart, resulting in oxygenated blood mixing with deoxygenated blood [9]. Anatomical variations of up to six PVs have been documented consistently with reported variations of up to four right-sided vessels with two standard left-sided vessels [1]. The highest quantity of PVs with corresponding ostia draining from a single lung found in the literature was 7 [10]. Across published literature in the English language, there are no reports of cases where the number of PVs deviated as drastically from published norms as the current case of 12 PVs, 10 of which connect to the right lung, with corresponding ostia that drain to the left atrium. 

Recently, there has been increased interest in PVs and their ostia due to their potential association with atrial fibrillation and ectopic heartbeats [11,12]. Knowledge of these anatomic variations is integral for patient safety when performing radiofrequency catheter ablation and catheter extirpation in patients with abnormal heart rhythms due to PV obstruction or hypertension [13]. The success of such treatments is dependent on having accurate information regarding PV anatomy, their measurements, and significant variation, which could substantially alter success and complication rates [11]. This case presentation of 12 supernumerary PVs intends to bring to light a rare anatomical variant and explore its potential clinical implications. 

## Case presentation

**Figure 1:**
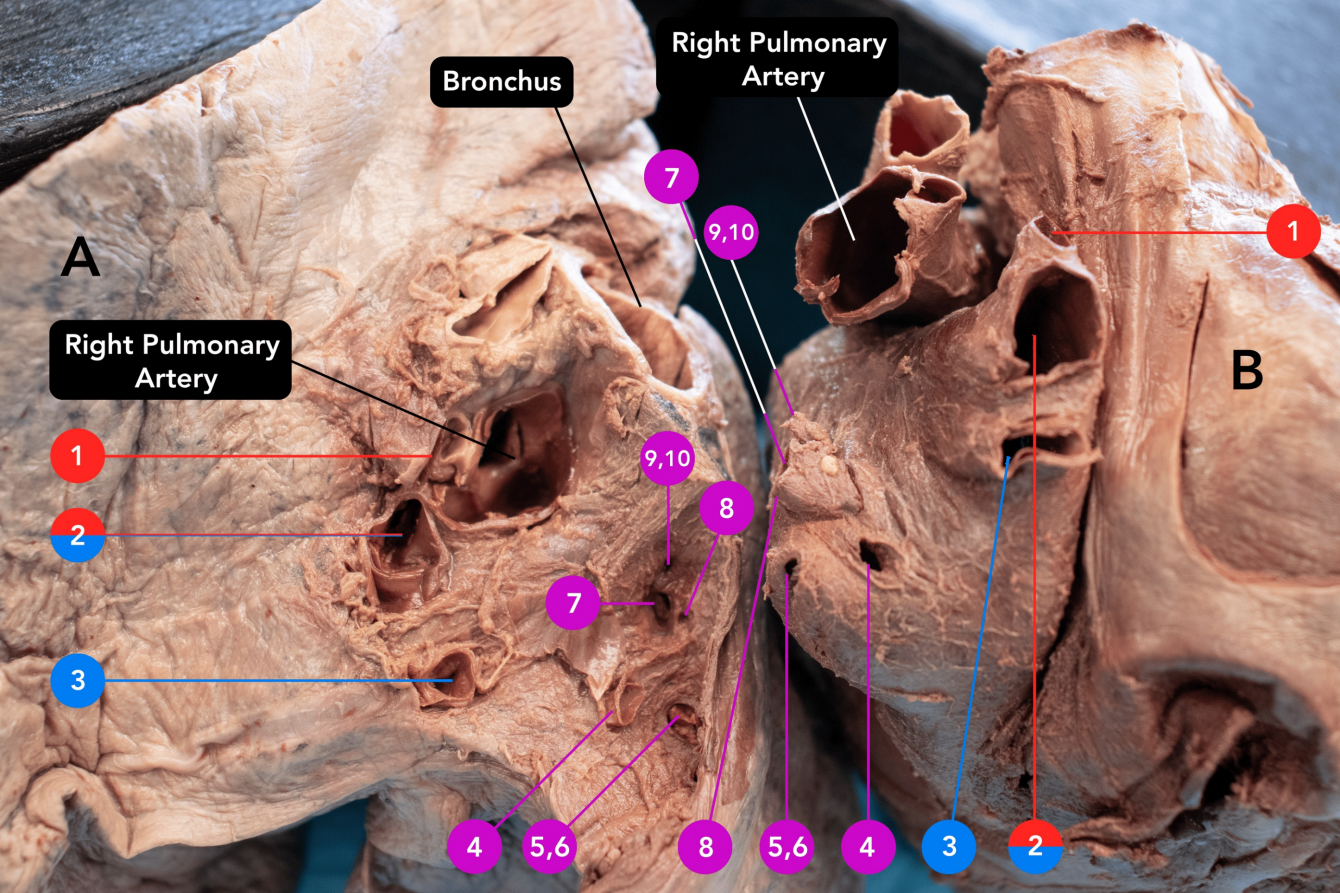
Pairing of accessory pulmonary veins between the right lung and the heart Pairing of accessory pulmonary veins between the right lung (A) and the heart (B). Each accessory vein links to a corresponding ostium. Each ostium of an accessory PV is indicated by a number labeled on the heart and lung. A red label indicates connection to the right upper lobe. A blue label indicates connection to the right middle lobe. A purple label indicates connection to the right lower lobe. Note that the right PV 2 was associated with both the right upper and right middle lobes. PV: Pulmonary vein

**Figure 2:**
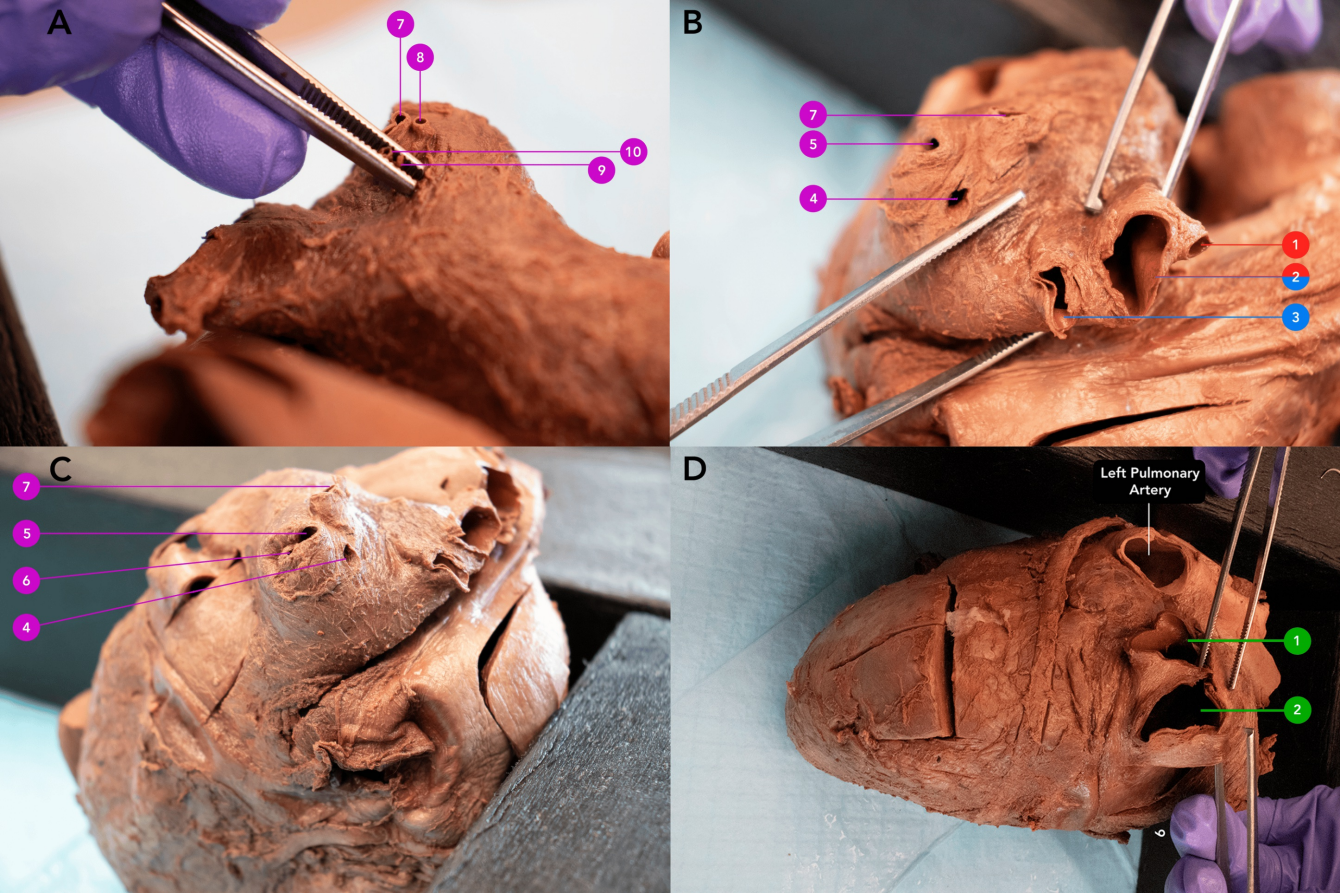
Views of aberrant pulmonary vein ostia opening into the left atrium Panel A: Right-sided accessory PVs (7, 8, 9, 10); Panel B: Right-sided accessory PVs (1, 2, 3, 4, 5, 7); Panel C. Right-sided accessory PVs (4, 5, 6, 7); Panel D. Normal left superior (1) and inferior PVs (2). A red label indicates connection to the right upper lobe. A blue label indicates connection to the right middle lobe. A purple label indicates connection to the right lower lobe. A green label indicates left-sided PVs. PV: Pulmonary vein

**Figure 3:**
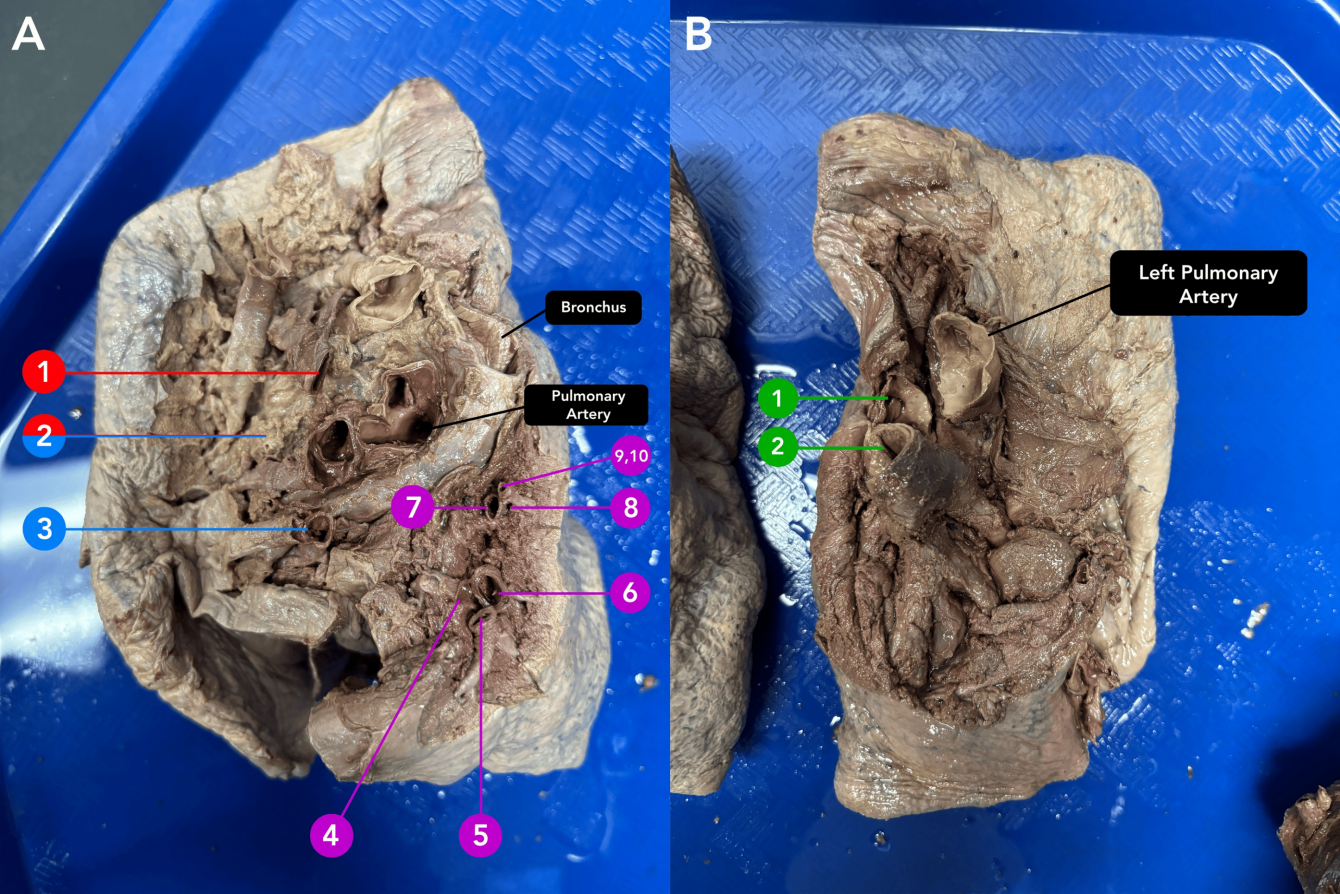
Views of aberrant pulmonary veins at the hila of the lungs Panel A. Right-sided PVs (1-10). Panel B. Left PVs (1,2). A red label indicates connection to the right upper lobe. A blue label indicates connection to the right middle lobe. A purple label indicates connection to the right lower lobe. A green label indicates left-sided PVs. PV: Pulmonary vein

An 87-year-old male body was received through the Saint Louis University Gift Body Program of the Center for Anatomical Science and Education (CASE) with a signed informed consent form from the donor. The CASE gift body program abides by all the rules set forth by the Uniform Anatomical Gift Act (UAGA). During routine dissection, the donor was found to have received multiple bypass grafts. This was later confirmed with a limited donor medical history. Upon removal of the heart and lungs, unusual variations were identified in the quantity of PV connected to the left atrium. Rather than the typical expected four PVs, the donor had 12 PVs (Figure 1). Each of the 12 PVs entered separately into the left atrium with its own corresponding ostia (Figure 2). The left lung presented the expected, normal anatomy with two PVs draining from the left lung (Figure 3). However, the right side of the left atrium displayed significant anomalous anatomy. Rather than the expected two PVs, 10 additional PVs were observed with a range in diameters of less than 1 mm to 20 mm (Figure 1, Table 1). 

**Table 1:** Axes, cross-sectional areas, and lobes drained by right and left PVs PV: Pulmonary vein

Name/ Location of PV	Short Axis (mm)	Long Axis (mm)	Cross-Sectional Area (mm^2^)	Total Area (mm^2^)	Lobes Drained	Lobar Area Drained (mm^2^)
Left PV 1	14	19	209	429	Left Upper	209
Left PV 2	14	20	220		Left Lower	220
Right PV 1	5	5	20	173	Right Upper & Right Middle	143
Right PV 2	11	11	95			
Right PV 3	6	6	28			
Right PV 4	3	2	5		Right Lower	30
Right PV 5	2	2	3			
Right PV 6	4	4	13			
Right PV 7	3	3	7			
Right PV 8	1	1	1			
Right PV 9	1	1	1			
Right PV 10	1	1	1			

Anatomically, the accessory PVs were separated into three distinct groups based on the lobes from which they drained blood. Through additional dissection of the right lung, the drainage of the right PVs 1-10 was traced and elucidated as follows: right PV 1 drained blood from the right upper lobe, right PV 3 drained the middle lobe, and right PVs 4-10 received the blood from the lower lobe (Figure 3). Right PV 2 was unique in that it drained both the upper and middle lobes. The horizontal fissure was present but did not fully divide the lobes. On the left lung, left PV 1 and PV 2 drained the left upper lobe and lower lobe, respectively. The lumen of each left and right PV can be seen in Figure 1. 

A digital caliper was used to measure the elliptically shaped lumen of each PV ostia on the heart. The total area was then calculated using the formula for the area of an ellipse (A = π * radius of the short axis * radius of the long axis). Each diameter measurement was rounded to the nearest millimeter (Table 1). Right PVs 1-3 drained the right upper and middle lobes, reflecting the expected drainage of a right superior PV. As such, the total drainage to the right upper and middle lobes was calculated to be 143 mm^2^. The lower lobe was drained by PVs 4-10, reflecting the drainage of the right inferior PV, with a total cross-sectional area of 30 mm^2^. Summing the cross-sectional area of each of the 10 right PVs, the total PV area draining from the right lung was 173 mm^2^ (Table 1). However, it is important to note that these measurements may represent a slight underestimation of the true vessel caliber due to vessel collapse or post-mortem artifact. 

Compared to the cross-sectional area of the right PVs, the normal PVs connecting to the left lung displayed a significantly greater cross-sectional area. Left PV 1 and left PV 2 cross-sectional areas were 209 mm^2^ and 220 mm^2^ respectively, totaling 429 mm^2^ (Table 1). 

## Discussion

PVs function to transfer freshly oxygenated blood from the lungs to the left atrium of the heart. Most individuals have four PVs, two on the left and two on the right; however, variations are present in about 30% of patients [14]. There has also been significant variability reported in the pulmonary vein diameter and distance to first bifurcation [7], which could potentially have clinical implications. While most cases of accessory PVs are benign [15], knowledge of anatomical PV abnormalities can provide accurate information to clinicians during the diagnosis of disease and is crucial for the safety and efficacy of interventional procedures performed on the heart. 

In this donor, the total cross-sectional area of the right PVs was 60% smaller compared to that of the left PVs. The relationship between these areas deviates from the expected values found in the literature. The mean diameters of each of the four typical PVs are normally asymmetric. Previous publications have found that right superior PVs have diameters within a 95% confidence interval of 11.4-12.4 mm with cross-sectional areas within a 95% confidence interval of 122.2-142.2 mm^2^, right inferior PVs have diameters between 12.3 and 13.1 mm (cross-sectional area = 134.5-150.8 mm^2^), left superior PVs have diameters between 9.6 and 10.5 mm (cross-sectional area = 98.6-114.4 mm^2^), and left inferior PVs have diameters between 9.0 and 9.9 mm (cross-sectional area = 91.4-104.6 mm^2^) [16]. Totaling these values, the right lung is drained by an average cross-sectional area of 274.8 mm^2^. In this donor, the right-sided cross-sectional area of 173 mm^2^ is smaller than expected. By decreasing this area, the resistance of blood flow was likely much greater, presumably increasing the pressure experienced in the right lung. These assumptions are made based on Poiseuille’s and Ohm’s laws, which outline the indirect relationship between vessel radius and resistance to flow and the direct relationship between resistance and pressure, respectively. Similar hemodynamics can be appreciated in other known vascular pathologies involving pulmonary vessel occlusion or narrowing, such as PV thrombosis or stenosis. As such, it is possible that the increased vessel resistance and pressure associated with the significantly reduced cross-sectional area could contribute to the development of cardiopulmonary pathologies, such as pulmonary hypertension, though it is unknown whether or not this applies to this donor. However, while the PV drainage area from the right lung is reduced, the drainage from the left lung is drastically increased. In total, the left lung is typically drained by a cross-sectional area of 204.5 mm^2^. In this donor, the left-sided cross-sectional area of 429 mm^2^ is significantly larger than the expected values. It is possible that this represents a reactive process compensating for the decreased flow from the right lung or a direct result of aberrant embryologic development. Interestingly, the total cross-sectional area of all PVs in this donor was 602 mm^2^, appreciably higher than the expected 479.3 mm^2^, a majority of which was attributed to the left PVs. Given the limited research behind supernumerary PVs and the lack of medical history available for our donor, it is unclear what the true etiology of this pulmonary venous anomaly is. However, with understanding of the embryologic development of the PVs, it likely represents a variation in the formation and incorporation of the common PV into the left atrium. 

Recent research identified that ectopic foci found in PVs play a prominent role in the pathogenesis of atrial arrhythmias [11,17], with a higher reported incidence of paroxysmal atrial fibrillation in patients with abnormal PV anatomy [12,18]. The major source of ectopic beats appears to be the muscular sleeves covering the outer surface of PVs, formed by extensions of the left atrial myocardium during embryologic development. Douglaset al. hypothesize that derangements in PV formation may contribute to electrical conduction and rhythm disorders [17]. As such, cardiac computed tomography and magnetic resonance imaging, often with angiography, are being used increasingly in both the planning and execution of catheter ablation procedures for atrial fibrillation in order to provide a map of the patient’s unique cardiopulmonary anatomy [19]. Radiofrequency catheter ablation can isolate the PV and ablate the focal area that is causing the arrhythmia [12]. This procedure can be made more difficult by uncertain catheter positioning within the PVs and difficulty in localizing the ectopic foci in the case of variable anatomy [20]. Therefore, though variant PV anatomy is often asymptomatic, it may assume importance in pre-interventional planning and assessing risk factors for certain cardiopulmonary diseases. This case brings to light an extreme variant of a known pulmonary venous anomaly with the intention of contributing to anatomical education and prompting discussion regarding its potential clinical implications. 

While the medical history available for this donor is limited due to the self-reported nature, it is notable for an unknown type of kidney surgery (1966), colon cancer surgery (1996), coronary bypass (2001), prostate cancer radiation (2005), and knee surgery (2012). His death at the age of 87 years was attributed to congestive heart failure and atherosclerotic heart disease as indicated on the death certificate. Notably, these complications did not result in an appreciably reduced life span, given that he lived longer than the average American life expectancy of 76.1 in 2021 [21]. 

## Conclusions

This case report detailing an individual with the highest reported number of supernumerary PVs elucidates the importance of knowledge of anatomical variants of PV circulation in both the educational and clinical settings. Though rare, supernumerary PVs have the potential to impact the management of cardiovascular complications such as atrial fibrillation, ectopic heartbeats, and heart failure. This represents a potential risk factor for patients that is not commonly assessed in current clinical practice. Population screening with routine imaging and electrophysiological mapping in patients with cardiovascular disease and atrial fibrillation, respectively, would be necessary in order to further elucidate the role of accessory PVs in cardiac function and explore their impact on patient prognosis and treatment. 
